# Unified translation repression mechanism for microRNAs and upstream AUGs

**DOI:** 10.1186/1471-2164-11-155

**Published:** 2010-03-05

**Authors:** Subramanian S Ajay, Brian D Athey, Inhan Lee

**Affiliations:** 1 Bioinformatics Graduate Program, University of Michigan, Ann Arbor, MI 48109, USA; 2 Department of Psychiatry, University of Michigan, Ann Arbor, MI 48109, USA; 3 Center for Computational Medicine and Biology, University of Michigan, Ann Arbor, MI 48109, USA; 4 miRcore, 2929 Plymouth Rd, Ann Arbor, MI 48105, USA; 5 Current address: Genome Technology Branch, National Human Genome Research Institute, National Institutes of Health, Bethesda, MD 20892, USA

## Abstract

**Background:**

MicroRNAs (miRNAs) are endogenous small RNAs that modulate gene expression at the post-transcriptional level by binding complementary sites in the 3'-UTR. In a recent genome-wide study reporting a new miRNA target class (miBridge), we identified and validated interactions between 5'-UTRs and miRNAs. Separately, upstream AUGs (uAUGs) in 5'-UTRs are known to regulate genes translationally without affecting mRNA levels, one of the mechanisms for miRNA-mediated repression.

**Results:**

Using sequence data from whole-genome cDNA alignments we identified 1418 uAUG sequences on the 5'-UTR that specifically interact with 3'-ends of conserved miRNAs. We computationally identified miRNAs that can target six genes through their uAUGs that were previously reported to suppress translation. We extended this meta-analysis by confirming expression of these miRNAs in cell-lines used in the uAUG studies. Similarly, seven members of the *KLF *family of genes containing uAUGs were computationally identified as interacting with several miRNAs. Using *KLF9 *as an example (whose protein expression is limited to brain tissue despite the mRNA being expressed ubiquitously), we show computationally that miRNAs expressed only in HeLa cells and not in neuroblastoma (N2A) cells can bind the uAUGs responsible for translation inhibition. Our computed results demonstrate that tissue- or cell-line specific repression of protein translation by uAUGs can be explained by the presence or absence of miRNAs that target these uAUG sequences. We propose that these uAUGs represent a subset of miRNA interaction sites on 5'-UTRs in miBridge, whereby a miRNA binding a uAUG hinders the progression of ribosome scanning the mRNA before it reaches the open reading frame (ORF).

**Conclusions:**

While both miRNAs and uAUGs are separately known to down-regulate protein expression, we show that they may be functionally related by identifying potential interactions through a sequence-specific binding mechanism. Using prior experimental evidence that shows uAUG effects on translation repression together with miRNA expression data specific to cell lines, we demonstrate through computational analysis that cell-specific down-regulation of protein expression (while maintaining mRNA levels) correlates well with the simultaneous presence of miRNA and target uAUG sequences in one cell type and not others, suggesting tissue-specific translation repression by miRNAs through uAUGs.

## Background

MicroRNAs (miRNA) are short 21-23 nt sequences that regulate gene expression post-transcriptionally [1,2]. Two processes, mRNA destabilization and translational repression, are believed to occur as a result of miRNA targeted gene regulation [3]. Many miRNA target prediction strategies rely on sequence matches between the miRNA seed region (positions 2-7 from the 5'-end) and well-conserved sites on the 3'-UTR [4,5]. Identification of several factors contributing to specificity of 3'-UTR target sites has helped improve target prediction methods [6]. However, not all target sites reside on the 3'-UTR; a few reports have shown that 5'-UTR and coding sequence (CDS) sites are functional as well [7-12].

Translation initiation in eukaryotes is postulated to follow the ribosome scanning model [13], possibly constrained by multiple cis-elements on the 5'-UTR such as secondary structure [14], 5'-terminal oligopyrimidine tracts [15] and upstream AUG (uAUG) nucleotides [16]. It is known that uAUGs cause a reduction in translational efficiency, therefore acting as a strong negative regulator of gene expression [13]. Comparative genomic analysis has revealed that uAUGs are conserved in mammalian 5'-UTRs to a greater extent than in other segments of mRNAs, genes harboring them mainly coding for transcription factors [17]. uAUGs may form alternative start sites forming upstream open reading frames (uORF), which are known to reduce efficiency of translation, possibly by translation of the uORF-encoded peptide [18]. It has been noted that a uAUG/uORF can inhibit translation independent of a downstream secondary structure or its position relative to other uAUGs before the main ORF [19,20].

Unlike the start codon of the main ORF, which in good initiation context is typically identified by the consensus Kozak sequence [21], many of the uAUGs are in sub-optimal context for translation [16]. Some groups have been able to assay for *in vitro*-translated uORFs [22,23], which are not, however, readily detectable unless fused to a reporter gene [24,25]. One study showed that translation repression is not dependent on the encoded peptide sequence [23], which suggests that the peptide action may be non-specific. Further, Kwon *et al. *demonstrated that addition of a synthetic peptide encoded by a uORF did not alter translation of the protein-coding gene even though the uORF on the 5'-UTR was able to repress translation [24].

Moreover, previous studies have reported that the uAUGs' effect on translation repression is specific to tissue type: though mRNAs containing uAUGs are expressed ubiquitously, the proteins are expressed only in specific tissues [19,26]. If indeed the translation of uORF limits downstream ORF translation, why does this repression occur only in certain cell-lines and tissues? There appears to be an additional mechanism of translation repression through uAUG other than upstream-encoded peptides.

Earlier, through computational analysis we discovered the presence of genome-wide sites on 5'-UTRs that interact with 3'-ends of miRNAs, a few of which were experimentally validated [12]. In this report we identify a subset of these miRNA interactions specific to the uAUG that occur preferentially through the 3'-end of the mature miRNA sequence. Based on our findings, we hypothesize that miRNAs expressed in one cell type but not in others may account for differences in protein expression in the cell types without changes in mRNA levels. Using miRNA expression data and results from prior work done with the *KLF9 *gene in HeLa and N2A cells, we demonstrate the validity of our hypothesis. Our results suggest the role of miRNAs in cases where uAUG confers tissue-specific protein expression of the target mRNA.

## Results

### uAUGs are potential miRNA target sites

**Figure 1:**
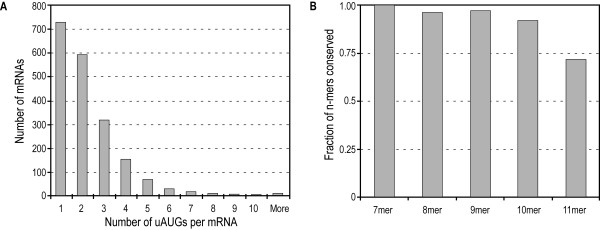
**Number of uAUGs in 5'-UTRs and their conservation**. **(A) **Distribution of uAUGs in human 5'-UTR sequences **(B) **Fraction of uAUG-containing n-mer sequences conserved in human and mouse 5'-UTRs.

An earlier study of excess conservation of uAUGs used a total of 1955 pairwise alignments of human and mouse 5'-UTR sequences [17]. The authors generated the alignments after careful pre-processing steps to remove any coding sequences that may have been mis-annotated as leader sequences. We used this well-curated alignment data to compile sequences containing uAUGs from human 5'-UTRs (see Methods), generating a total of 4009 11-mers centered on uAUG. The number of uAUGs per 5'-UTR ranges from one to 20, with 68% of the 1955 human 5'-UTRs containing at most two (Fig. 1A). Churbanov *et al. *[17] showed that upstream AUG triplets were conserved more than any other on the 5'-UTR. In order to investigate conservation patterns around the uAUG we looked at the identities of nucleotides in subsequences of 11-mers that were extracted. The uAUG sequences appear to be highly conserved between both human and mouse UTRs, with all 7-mers having 100% identities and roughly 70% of 11-mers being conserved (Fig. 1B). This indicates that the nucleotides surrounding uAUGs are well conserved between the two mammalian 5'-UTRs.

Mature human miRNA sequences (miRBase, version 11.0) [27] were downloaded and categorized as conserved (471 sequences) or non-conserved (206 sequences) miRNAs (see Methods). To reveal preferential interaction with any portion of the miRNA we split each sequence into its 5'- and 3'-ends, the former containing the seed region. We then looked for sequence matches between miRNA ends and the uAUG-containing sequences generated. This was done in two steps: 1) a thermodynamics-based search using RNAhybrid [28] with a ΔG cutoff ≤ -14 kcal mol^-1 ^followed by 2) a filter step to look for 7 or more consecutive matches with zero or one GU wobbles. To control for spurious hits, the number of interacting pairs was compared to the number obtained after shuffling the mature miRNAs sequences and repeating the search procedure.

**Figure 2:**
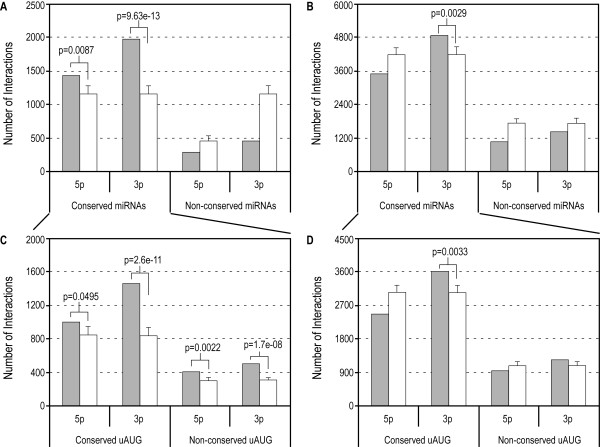
**Interaction of miRNAs with uAUG sequences**. Each predicted interaction is characterized by a 7-mer consecutive match between the indicated half of mature miRNA (5p and 3p for the 5'- and 3'-end respectively) and uAUG sequence with ΔG_37 _≤ -14 kcal mol^-1^. Grey bars represent actual counts and white bars represent average number of counts over 1000 repetitions of miRNA shuffling. Error bars represent the standard deviations. Significant outcomes are indicated with the corresponding p-values. **(A, B) **Number of interactions between uAUG sequences (4009 in total) and conserved and non-conserved miRNAs (471 and 206 in total respectively) without GU wobbles (A) and with at most one GU wobble (B). **(C, D) **Number of interactions between conserved miRNAs and uAUG sequences (2935 conserved and 1074 non-conserved) without GU wobbles (C) and with at most one GU wobble (D).

We observed many predicted interactions between uAUG sequences and the two miRNA ends, characterized by a dependency on conservation of miRNAs. Only conserved miRNAs showed a significant number of interactions while non-conserved miRNAs were no better than their shuffled cohorts (Fig 2A and 2B). There were a number of 7-mer Watson-Crick complementary matches between the 5'-ends of conserved miRNAs and uAUG sequences (Fig 2A). Interestingly, there seemed to be a greater number of such interactions at the 3'-ends (Fig. 2A), which suggests a preference for pairing between uAUGs and 3'-ends. These interactions arose from 46 conserved miRNAs and 263 unique uAUG motifs of length 7 or more (Table 1). Further, when we included at most one GU wobble the only significant result that persisted was the interaction with the 3'-ends of conserved miRNAs (Fig. 2B). Previously, we conducted a genome-wide motif study of 5'-UTRs and 3'-UTRs and observed a starkly similar propensity for interaction between 5'-UTRs and 3'-ends of miRNAs, few of which were validated [12]. Another study reported similar observations wherein 5'-UTR and coding regions participate in binding the 3'-end of the highly conserved miRNA, *let-7 *[10]. The preference for interaction with 3'-ends suggests the role of non-seed region matches in the 5'-UTR, while seed-region matches prevail in the 3'-UTR. This may explain the fact that there are very few known endogenous targets on the 5'-UTR that exhibit seed-matches [29]. We conducted a brief GO-term investigation into the nature of genes containing the uAUGs listed in Table 1. Out of a total 1071 genes that contained these uAUGs we were able to retrieve annotations for 678 genes. The majority of these 678 were found to be involved in transcription factor activity (See Additional file 1).

**Table 1:** MicroRNAs predicted to interact with uAUG-containing motifs

miRNA*	uAUG-containing motifs^§^
hsa-let-7d	AACUAUG, ACUAUGCAA, CUAUGCAAC

hsa-miR-130a/b	AUGCCCU

hsa-miR-132	GACCAUGGCU

hsa-miR-146a	ACCCAUGG, CCCAUGGAA

hsa-miR-146b-5p	GCCUAUGG, CCUAUGGAA

hsa-miR-194	CCACAUGGA, ACAUGGAG

hsa-miR-199a-3p	ACCAAUGUG

hsa-miR-202	UCCCAUGC, CCCAUGCC

hsa-miR-219-2-3p	ACAGAUGU, CAGAUGUCC, AGAUGUCCA

hsa-miR-297	GCACAUGC

hsa-miR-299-5p	AUGUAUGUGGG

hsa-miR-31	GCUAUGCCA, CUAUGCCAG

hsa-miR-324-5p	ACCAAUGCC, CAAUGCCC

hsa-miR-33a/b	GCAAUGCA, CAAUGCAA, AUGCAAC

hsa-miR-34b	AUGGCAG

hsa-miR-363	ACAGAUGGA, AGAUGGAU, CAGAUGGAU, GAUGGAU

hsa-miR-376b	AACAUGGAUU

hsa-miR-380	AAGAUGUGG, AGAUGUGGA, GAUGUGGA

hsa-miR-431	GCAUGACG, CAUGACGG

hsa-miR-432	CCCAAUGA, CCAAUGAC

hsa-miR-448	AUGGGAC

hsa-miR-450b-3p	AUGGAUGCA, GGAUGCAA

hsa-miR-455-3p	GUAUAUGC, AUAUGCC

hsa-miR-455-5p	CGAUGUAG, GAUGUAGU

hsa-miR-487a	CUGGAUGUC

hsa-miR-487b	GUGGAUGA, UGGAUGAC

hsa-miR-490-3p	CAGCAUGGAG, AGCAUGGAGU

hsa-miR-491-5p	CCUCAUGGAAG

hsa-miR-513b	AUAAAUGACA, AUGACAC

hsa-miR-556-3p	AAAGAUGAGC, AGAUGAGCU

hsa-miR-562	GCAAAUGGU

hsa-miR-580	CCUAAUGA, AUGAUUC

hsa-miR-583	UAAUGGGA, AAUGGGAC

hsa-miR-598	GACGAUGAC, ACGAUGACA

hsa-miR-609	AGAGAUGAG, GAGAUGAGA

hsa-miR-654-3p	GGUGAUGGU

hsa-miR-654-5p	GCACAUG, ACAUGUUCU

hsa-miR-767-3p	AACCAUGGG

hsa-miR-802	AAGGAUGAAU

hsa-miR-887	CGGGAUGG

hsa-miR-889	AAUGGUUG

hsa-miR-890	ACUGAUGC, CUGAUGCC

hsa-miR-942	CACAUGGCC, ACAUGGCCA

hsa-miR-944	UCCGAUG

* The 46 miRNAs represent conserved miRNAs

^§^Only the portion of uAUG11-mer that interacts with the 3'-end of miRNAs without a GU wobble is presented. If a miRNA matches a uAUG sequence and its subsequence(s), only the longest form is presented.

As nearly 75% of the 11-mers were found to be conserved between human and mouse 5'-UTRs (2935 out of 4009), we investigated if the interactions with conserved miRNAs were a function of uAUG sequence conservation. Results showed no dependence on uAUG conservation when not allowing GU wobbles (Fig. 2C). However, when allowing at most one GU wobble, only conserved uAUGs exhibited significant interactions with 3'-ends of miRNAs (Fig. 2D).

The above results indicate that uAUGs may participate in highly sequence-specific Watson-Crick base-pairing with miRNAs, particularly towards the 3'-ends. The fact that inclusion of a GU wobble still resulted in a significant number of interactions between the 3'-ends and uAUGs suggests functionality.

### Expressed miRNAs may bind endogenous uAUG sites

The analyses that follow are based on experiments with genes that contain uAUGs in their 5'-UTRs, drawing upon sequence data and results from previous experiments that attribute translational repression to the uAUGs. We also used miRNA expression evidence from several sources - these references are consolidated in the form of meta-data (Table 2). We extracted 11-mer sequences containing uAUGs for these genes and looked for interactions with conserved miRNAs using the search strategy outlined above. Based on the observations in Fig. 2A and 2B, we allowed one GU wobble for interactions with the 3'-end and none with the 5'-end. Many of the genes contain multiple uAUGs/uORFs that have different inhibitory effects on translation. We assigned discrete values to these uAUGs that reflect their ability to repress expression of a downstream reporter. These were obtained by comparing the effect of the uAUG either on a control construct or on a construct where the uAUG under consideration is mutated or deleted. The values range from 1× to 6×, where 1× indicates that the uAUG is least repressive or does not show any effect. Sequences that limit the expression of reporter to half or one-third the control or mutant case are assigned a value of 2× or 3× respectively, and so on.

**Table 2:** Genes used in analysis along with references

Gene	Evidence showing translational control by uAUG	miRNA expression evidence used for analysis
*KLF9/BTEB1*	[19]	[41-43]*

*KLF13/RFLAT-1*	[26]	[41,42,44,45]

*MOR*	[25]	[41,42]

*CHOP*	[46]	[41,42]

*MDM2*	[20]	[41,42]

*ADH5/FDH*	[24]	[41,42]

* miRNAs expressed in mouse N2A cells are not listed in ref. 43 (unpublished data). They were obtained through personal communication with the authors.

We not only observed complementary matches with conserved miRNA sequences but also confirmed the presence of many of the predicted miRNAs in cell-lines where repression was observed (Table 3). There also appears to be an association between repressive strength of uAUGs and miRNA target predictions. Two uAUGs that have little or no effect on repression are indicated by '1×' in Table 3, lacking miRNA interaction sites. Conversely, uAUGs with strong repressive potential (2×-6×) are complementary to expressed miRNAs except for the first uAUG in the ADH5/FDH gene and second uAUG of the KLF13 gene, where expressions of the predicted miRNAs have not been detected. In cases where there is more than one uAUG, more than one miRNA may act in a combinatorial manner to produce a net repressive effect. This is in line with observations of interactions between many miRNAs and single 3'-UTR [30]. These observations suggest that some of the uAUG sequences are miRNA-specific and functional target sites.

**Table 3:** Genes containing uAUGs predicted to interact with expressed miRNAs

Gene	uAUG^†^	Cell line used in experiments	miRNAs predicted to interact^§^	Evidence of miRNA expression^‡^
*MOR*	gcccAUGcucc (1×)	HEK293	*hsa-miR-146a (3')*	No
			hsa-miR-202 (3')	No
	ggggAUGcuaa (2×)		hsa-miR-324-5p (5')	Yes[42]
			hsa-miR-517b (5')	Yes[42]
	aaggAUGcgcc (3×)		*hsa-miR-323-5p (3')*	No
			hsa-miR-324-5p (5')	Yes[42]
			*hsa-miR-450b-3p (3')*	No

*CHOP*	uaucAUGuuaa (1×)	HeLa	None	
	aaagAUGagcg (6×)		hsa-miR-574-3p (5')	Yes[41,42]
			hsa-miR-556-3p (3')	No
	gcagAUGugcu (2×)		hsa-miR-219-2-3p (3')	No*

*MDM2*	aaagAUGgagc (3×)	HeLa	hsa-miR-363 (3')	Yes[42]
	uggaAUGaucc (1×)		None	

*ADH5/FDH*	gcccAUGccuc (4×)	HeLa	*hsa-miR-146a (3')*	No
			hsa-miR-202 (3')	No
	ccggAUGucag (4×)		*hsa-miR-219-1-3p (3')*	No*
			*hsa-miR-219-2-3p (3')*	No*
			hsa-miR-487a (3')	No
			hsa-miR-489 (5')	No

*KLF13*	cacaAUGcgcg^# ^(1×)	Jurkat	*hsa-miR-323-5p (3')*	No
			hsa-miR-103 (5')	Yes[41]
			hsa-miR-107 (5')	Yes[41]
			hsa-miR-33a (5')	Yes[44,45]
	ccccAUGcgcu (2×)		hsa-miR-586 (5')	No
			hsa-miR-202 (3')	No
	gcggAUGcgcg (2×)		*hsa-miR-450b-3p (3')*	No
			hsa-miR-324-5p (5')	Yes[41,44]

^† ^uAUGs shown in caps. Strength of translation repression (1×-6×) was deduced by comparing the effect of the uAUG either to a control construct or to a construct where the uAUG under consideration is mutated or deleted.

^# ^uAUG not present in the GenBank entry but used in reporter constructs [26].

^§ ^Numbers in parentheses indicate the miRNA end predicted to interact. miRNAs in italics indicate matches with one GU wobble.

^‡ ^Reference for evidence of expression.

*Expression of mature miR-219, which corresponds to the 5p arm of the precursor, was detected by Chen *et al.*, but that of 3p was not assayed for on the microarray [42].

### KLF genes are probable 5'-UTR miRNA targets

Kruppel-like factors (KLFs) are transcriptional regulators that contain a characteristic zinc-finger domain and are known to play a role in differentiation and other cellular events [31,32]. There are as many as 15 members in this family, seven of them containing at least one uAUG. Using the criteria set above we identified 7-mer matches between uAUG-containing sequences and miRNAs in all seven of these genes (Table 4). Two of these, *KLF9 *and *KLF13*, also called *BTEB1 *and *RFLAT-1 *respectively, are known to be translationally regulated by uAUGs in their 5'-UTRs [19,26]. The uAUGs in these two genes have been implicated in cell-specific control of protein expression though their respective transcripts are present in many other tissues, suggesting a post-transcriptional mechanism of gene regulation [19,26].

**Table 4:** uAUGs from members of the KLF family predicted to interact with conserved miRNAs

KLF Gene^§^	uAUG^†^	miRNAs predicted to interact^‡^
*KLF6* (NM_001300)	uugcAUGaaac	*hsa-miR-93 (3')*

*KLF7* (NM_003709)	cuggAUGccuc	hsa-miR-450b-3p (3'), hsa-miR-487a (3')
	cuggAUGucug	*hsa-miR-450b-3p (3')*, hsa-miR-487a (3')

*KLF8* (NM_007250)	cucuAUGauuc	hsa-miR-376a (5'), hsa-miR-376b (5'),
		hsa-miR-376c (5')
	cuuuAUGuuca	None
	gaggAUGggug	*hsa-miR-331-3p (3')*, *hsa-miR-363 (3')*,
		*hsa-miR-802 (3')*, *hsa-miR-99b (5')*
	uuggAUGcuug	hsa-miR-450b-3p (3')
	cgcuAUGucag	*hsa-miR-31 (3')*
	cagaAUGgggc	*hsa-miR-448 (3')*, *hsa-miR-583 (3')*
		*hsa-miR-136 (5')*
	gaguAUGagcc	hsa-miR-767-3p (5')
	cggcAUGaguu	hsa-miR-574-3p (5')

*KLF10* (NM_001032282, isoform a)	gauuAUGcaau	hsa-let-7d (3'), hsa-miR-153 (5')
	agcaAUGgcuc	hsa-miR-160 (5')
	caucAUGcauu	None
	aagaAUGuuuu	None
	uuuaAUGgaaa	None

*KLF12* (NM_007249)	aucaAUGugac	hsa-miR-199a-3p (3')
		hsa-miR-23a (5')
		hsa-miR-23b (5')
	acaaAUGgaug	hsa-miR-136 (5')
	auggAUGaaug	hsa-miR-450b-3p (3')
		hsa-miR-487b (3')
		hsa-miR-802 (3')
	augaAUGaaua	None

^§ ^KLF13 and KLF9 are presented along with miRNA expression data in Table 3 and 5, respectively.

^† ^uAUGs are shown in caps.

^‡ ^Numbers in parentheses indicate the miRNA end predicted to interact. miRNAs in italics indicate matches with one GU wobble.

Specifically, protein expression of *KLF9*, whose 5'-UTR contains 10 uAUGs, is limited to brain tissue though its mRNA is expressed ubiquitously [19]. The 5'-UTR, particularly the portion containing uAUGs 6 and 7, suppressed reporter gene translation in HeLa cells but not in mouse neuroblastoma (N2A) cells [19]. This observation was even more intriguing because peptides from the two uORFs starting from uAUG6 and uAUG7 have not been detected [19]. Similarly, though *KLF13 *mRNA is expressed in multiple tissues, protein expression was only detected in adult spleen and lung tissues [33]. While *KLF13 *mRNA levels are constant throughout T-cell activation, KLF13 protein is only expressed later on in the activation process [26]. Presence of several uAUGs in its 5'-UTR down-regulated translation of the reporter gene in Jurkat T-cells and, to a lesser degree, in HEK293 cells [26].

We decided to focus our analysis on *KLF9 *uAUGs since the effects of wild-type and mutant constructs used to elucidate the roles of uAUGs were demonstrated in both cell-lines relevant to tissue specificity. We extracted uAUG 11-mers from the *KLF9 *5'-UTR sequence used in the experimental study [19] and searched for interactions with both ends of conserved miRNAs. Since the 5'-UTR study for *KLF9 *was also done in the mouse neuroblastoma (N2A) cell line, we used both mouse and human miRNAs in the analysis. All uAUGs except uAUG5 and uAUG8 interacted with at least one miRNA (Table 5). The ninth uAUG was predicted to interact with as many as five miRNAs. Most of these predicted miRNAs are expressed in HeLa cells but not in N2A cells, including those that match uAUG6 and uAUG7. Out of 26 human miRNAs predicted to interact with uAUGs (Table 5) 16 are reported to be expressed in HeLa cells, whereas out of 18 mouse miRNAs predicted only 5 are reported to be expressed in N2A cells.

**Table 5:** *KLF9 *uAUGs predicted to interact with miRNAs in HeLa cells

	uAUG^§^	miRNAs predicted to interact^†^	Evidence of miRNA expression^‡^	
			
			HeLa	N2A
1	cauaAUGgggu	hsa-miR-583 (3')	Yes[42]	---
		*hsa-miR-490-3p (3')*	---	---
		*mmu-miR-490 (3')*	---	---

2	aaagAUGuguc	miR-380 (3')	Yes[42]	---
		*hsa-miR-576-5p (3')*	Yes[42]	---

3	gccaAUGccag	*miR-16 (3')*	Yes[41,42]	Yes[41,43]
		hsa-miR-31 (3')	Yes[41,42]	**---**
		miR-324-5p (3')	Yes[41,42]	---

4	aaagAUGuguc	miR-380 (3')	Yes[42]	**---**
		*hsa-miR-576-5p (3')*	Yes[42]	**---**

5	uuaaAUGucag	None	**---**	**---**

6	cgugAUGggau	*miR-448 (3')*	---	---
		*hsa-miR-583 (3')*	Yes[42]	---
		*hsa-miR-609 (3')*	Yes[42]	---
		miR-654-3p (3')	---	---
		hsa-miR-605 (5')	---	---
		*mmu-miR-325 (3')*	---	---

m6	cgugA**A**Gggau	hsa-miR-491-3p (3')	---	---
		miR-188-5p (5')	Yes[42]	---
		*hsa-miR-211 (3')*	---	---
		*hsa-miR-520 h (3')*	---	---
		mmu-miR-712 (5')	---	---
		mmu-miR-343 (5')	---	---

7	gagaAUGccgg	*hsa-miR-31 (3')*	Yes[41,42]	---

m7	gagaA**A**Gccgg	*mmu-miR-505 (3')*	---	---

8	gugaAUGuccu	None	---	---

9	guggAUGcugc	hsa-miR-450b-3p (3')	---	---
		miR-487b (3')	---	Yes[43]
		miR-103 (5')	Yes[42]	Yes[43]
		miR-107 (5')	Yes[42]	Yes[43]
		miR-338-3p (5')	Yes[41,42]	---
		mmu-miR-376b (3')	---	Yes[43]
		*mmu-miR-450a-3p (3')*	---	---

10	aaagAUGaggg	hsa-miR-556-3p (3'),	---	---
		hsa-miR-609 (3')	Yes[42]	---

^§ ^uAUG shown in caps, mutated sequences prefixed with letter 'm', and mutated positions shown in bold.

^† ^Three letter species codes (hsa/mmu) are indicated only when one sequence interacts and omitted if both interact. Numbers in parentheses indicate the miRNA end predicted to interact. miRNAs in italics indicate matches with one GU wobble.

^‡ ^Reference for evidence of expression.

Regulatory roles of each uAUG/uORF may be studied by mutating one or more of the uAUGs to mitigate repression. In the case of *KLF9*, mutation of uAUG6 or 7 or both relieved translation repression [19]. However, uAUG6 inhibits translation to a greater extent compared to uAUG7, the translation efficiency of the uAUG6 mutant construct being 5 times that of the wild-type construct compared to a two-fold increase for the uAUG7 mutant, based on figure seven from Imataka *et al. *[19]. Interestingly, five human miRNAs are predicted to interact with uAUG6, of which two are expressed in the HeLa cell lines and none in N2A cells (Table 5 and Additional file 2). Only one expressed miRNA, hsa-miR-31, is predicted to bind uAUG7. If these two uAUGs are indeed miRNA interaction sites, their mutation should presumably eliminate interactions with the miRNAs predicted in Table 5. To test this assumption, we repeated the analysis using mutated uAUG sequences that had been shown to relieve translational repression. When mutated, uAUGs implicated in mediation of translation repression in *KLF9 *showed fewer predicted interactions with miRNAs (Table 5, sequences m6 and m7) compared to wild-type sequences. Moreover, there was little evidence for expression of miRNAs matching mutated uAUG sequences.

## Discussion

Though uAUGs are known to act in post-transcriptional control of gene expression, there is no clear account of the mechanism involved when differences in activity of uAUGs exist across cell or tissue types. While studying uAUGs and miRNAs independent of one another, researchers observed that uAUGs affect gene expression by reducing protein levels while maintaining mRNA levels, just as with miRNA-mediated gene regulation.

Target sites for miRNAs have conventionally been thought to reside on conserved regions of the 3'-UTR and are predicted to bind the seed-region of a miRNA, while 5'-UTRs are thought to lack them [5,29]. Using a combination of thermodynamic and sequence-based searches, we found many uAUG sites on the 5'-UTR that are predicted to interact with miRNAs. Interactions with uAUGs were, however, restricted to conserved miRNAs, as we found no significant interactions with non-conserved miRNAs. A likely reason might be that exon sequences, which also harbor uAUGs, are under selective pressure, causing conserved miRNAs to also evolve with them while non-conserved miRNAs are under no such constraint. On a genome-wide scale it was similarly noted that interactions with 5'-UTR sequences came mainly from conserved miRNAs [12]. Though both ends of conserved miRNAs exhibited a significant number of interactions, we found a propensity for 3'-end interactions with uAUGs. These possibly constitute a subset of many such interactions identified earlier that were shown, using miRNAs and genes of interest, to cause repression [12]. Forman *et al. *have also shown *in silico *that a well-conserved miRNA, let-7, is predicted to base-pair with the 5'-UTRs through the remainder of the miRNA apart from the seed portion [10]. The signal-to-noise ratio observed in the interaction between uAUG motifs and miRNAs surpassed those in our genome-wide motif study, thereby suggesting the importance of this interaction. Based on this evidence, we hypothesized that the overlap in miRNA and uAUG function may arise from underlying sequence-specific interactions.

Examining many genes where uAUGs have regulatory properties, we demonstrated the connection between uAUG-mediated repression and the likelihood that they serve as binding sites for conserved miRNAs. miRNA expression data support this link, confirming the presence of miRNAs in cell-lines where reporter translation is affected by uAUGs. Further, we predict that many uAUGs in the *KLF *family of genes are miRNA-binding sites. Two uAUGs in the well-studied *KLF9 *are proven down-regulators of protein expression, with regulation observed only in HeLa cells. Many miRNAs likely to interact with these two sequences were found to be expressed in the HeLa and not in N2A cells, where regulation was not observed.

As mentioned in a previous study and also demonstrated by the GO-term analysis in our results, many genes that contain uAUGs are transcription factors [17]. Two reports show that several miRNAs and transcription factors in *C. elegans *and mammals are involved in feedback circuits [34,35]. Expanding these analyses to include transcription factors containing uAUGs in the 5'-UTRs might reveal more such miRNA-transcription factor regulatory networks.

Several other pieces of evidence point to the possible interaction between miRNAs and uAUGs on the 5'-UTRs. Orom *et al. *showed that miR-10a binds sequences downstream of a 5'-oligopyrimidine tract (5'-TOP) on *RPS16*, a gene encoding a ribosomal protein, to regulate its translation [9]. This exact binding site on the 5'-UTR was thought to be responsible for conferring cell-specific translational regulation [15]. Taken together with these findings, our results suggest that miRNAs can also interact with uAUG sequences and confer tissue specificity. This would constitute a unifying mechanism of translation repression for miRNAs and uAUGs. We specifically propose that the interaction of miRNAs with uAUGs may impede the progress of the scanning 40S ribosome subunit. Through reporter gene experiments we have shown that miR-34a can induce translation repression by binding to the 5'-UTR of its predicted target (*AXIN2*) in the absence of the 3'-UTR [12]. We also noted that repression was much higher when both UTRs were present. Based on these results we envision two very plausible scenarios: a) repression caused by binding of the 3'-end of miRNAs uAUGs in the 5'-UTR independent of a separate miRNA molecule that may bind to the 3'-UTR, or b) synergistic repression by both the seed region and 3'-end of miRNA due to simultaneous action of on the 3'-UTR and 5'-UTR, respectively. Interestingly, primer extension (toeprint) analysis reveals the presence of a 40S ribosomal subunit at the start codon on miRNA-repressed mRNAs [36]. The same technique also reveals stalling of ribosomes in the vicinity of uAUGs [24,25,37]. Furthermore, Ago2, a member of the Argonaute family of proteins [38,39] and a component of the functional micro-ribonucleoprotein (miRNP) complex, was found to co-sediment with 40S-containing complexes [36]. These facts indicate that miRNAs associated with miRNPs may recognize uAUG sequences as target sites and prevent translation.

## Conclusions

In this manuscript we present observations that suggest a miRNA role in translational control by uAUG cis-elements on the 5'-UTR. Specifically, we identified many interactions between uAUG sequences and conserved miRNAs which suggest a sequence-specific binding mechanism between these post-transcriptional regulatory factors. We also presented evidence to show that miRNAs possibly bind to uAUGs that inhibit translation of downstream reporters in cells where the miRNAs are expressed, thus explaining differential control. This expands the range of probable miRNA targets to include many endogenous sites on the 5'-UTR.

Our current knowledge has limited us to think of miRNAs and uAUGs as distinct regulatory mechanisms. While distinct functions of miRNAs or uAUGs are found in other contexts, our study unifies them as a single translational repression phenomenon whereby uAUGs act as miRNA target sites and translation is hindered.

## Methods

### uAUG sequences

Pairwise alignments between 5'-UTRs of mammalian human and mouse cDNAs were downloaded from the ftp site listed in Churbanov *et al. *[17]. From each alignment we extracted uAUG 11-mer sequences from the human 5'-UTR beginning at position -4 and ending at position +7, with the 'A' being designated as +1 (e.g. NNNNAUGNNNN, where N is any nucleotide). Sequences of length 7 to 10 nt (e.g. AUGNNNN, NNNNAUGN, etc.) were considered when the uAUG appears towards the beginning or end of an alignment. Only uAUG sequences sharing 100% identity with the mouse homolog were categorized as conserved while others were considered as non-conserved uAUGs. Experimentally characterized uAUG sequences in Table 3 were obtained from the references listed in Table 2. For the *KLF *family of genes in Table 4, uAUG sequences were extracted from the 5'-UTR portions of the full RefSeq mRNA.

### MicroRNA sequences

For the motif analysis, mature miRNA sequences were downloaded from miRBase (version 11.0) [27]. miRNAs present in at least one other species (e.g. hsa-let-7d and mmu-let-7d), irrespective of conservation at the nucleotide level, were categorized as conserved miRNAs (471 in total) and others as non-conserved miRNAs (206 in total). miRNAs were then split into their 5'- and 3'-halves to check for any preferential interaction with one end or the other.

### Sequence complementarity search

A two-step strategy was employed in looking for matches between uAUG 11-mers and miRNA sequences. First, the thermodynamic search program RNAhybrid [28] was used with-e option (ΔG) set to ≤ -14 kcal mol^-1^. Next, hits with at least seven consecutive nucleotide matches were selected.

### Shuffling procedure and significance testing

miRNAs were shuffled in order to keep the nucleotide composition of the sequences intact. The search strategy above was repeated over 1000 shuffling iterations and the average number of interactions was calculated. The resulting distribution of number of interactions was assumed to be normal and significance calculated using a Z-test.

### GO-term analysis

We used the Cytoscape plugin for BiNGO [40] to determine the molecular functions in *H*.*sapiens *that are over-represented in the set of genes that contain uAUGs from Table 1. We filtered out automatic annotations (evidence code: IEA) before beginning the analysis and used the default settings for all other options provided by the software package.

### miRNA expression

For miRNAs from Landgraf *et al.*'s study [41], we used their web visualization tool to assess the presence or absence of miRNAs in a given cell-line. For data from Chen *et al.*'s study [42], we used a p-value cutoff of 0.01 to report the miRNA as expressed. We obtained expression evidence for miRNAs of interest in N2A cells from Hohjoh *et al.*'s [43] study through personal communication. Expression data from Lawrie *et al.*'s [44] and Takada *et al.*'s [45] studies were obtained directly from the manuscripts and supplementary information.

## Competing interests

Publication of this paper may draw attention to miRcore, a newly founded non-profit organization supporting miRNA research.

## Authors' contributions

SSA designed the study, acquired sequence and expression data, performed data analysis and wrote the manuscript. BDA helped with data analysis and in drafting the manuscript. IL designed the study, performed data analysis, and wrote the manuscript. All authors have read and approved this manuscript.

## Supplementary Material

Additional file 1 **Genes containing uAUGs that do/do not interact with 3'-ends of conserved miRNAs**. GO-term analysis for two categories of genes that contain uAUGs. The first category consists of genes with uAUGs that are predicted to interact with 3'-ends of conserved miRNAs (likely targets). The second category of genes contains uAUGs but shows no such interactions. Click here for file

Additional file 2 **Predicted interactions between uAUG 6 and 7 (Table 5) of *KLF9 *and conserved miRNAs**. uAUG6 and uAUG7 are thought to be responsible for limiting translation of *KLF9 *in HeLa cells but not in N2A. Predicted binding between both ends of conserved miRNAs in Table 5 and the two uAUGs are shown. Click here for file

## References

[B1] Bartel DP MicroRNAs: genomics, biogenesis, mechanism, and function Cell 2004 116 2 281 297 10.1016/S0092-8674(04)00045-5 14744438

[B2] Kim VN MicroRNA biogenesis: coordinated cropping and dicing Nat Rev Mol Cell Biol 2005 6 5 376 385 10.1038/nrm1644 15852042

[B3] Filipowicz W Bhattacharyya SN Sonenberg N Mechanisms of post-transcriptional regulation by microRNAs: are the answers in sight? Nat Rev Genet 2008 9 2 102 114 10.1038/nrg2290 18197166

[B4] Brennecke J Stark A Russell RB Cohen SM Principles of microRNA-target recognition PLoS Biol 2005 3 3 e85 10.1371/journal.pbio.0030085 15723116 PMC1043860

[B5] Lewis BP Burge CB Bartel DP Conserved seed pairing, often flanked by adenosines, indicates that thousands of human genes are microRNA targets Cell 2005 120 1 15 20 10.1016/j.cell.2004.12.035 15652477

[B6] Grimson A Farh KK Johnston WK Garrett-Engele P Lim LP Bartel DP MicroRNA targeting specificity in mammals: determinants beyond seed pairing Mol Cell 2007 27 1 91 105 10.1016/j.molcel.2007.06.017 17612493 PMC3800283

[B7] Jopling CL Yi M Lancaster AM Lemon SM Sarnow P Modulation of hepatitis C virus RNA abundance by a liver-specific MicroRNA Science 2005 309 5740 1577 1581 10.1126/science.1113329 16141076

[B8] Lytle JR Yario TA Steitz JA Target mRNAs are repressed as efficiently by microRNA-binding sites in the 5' UTR as in the 3' UTR Proc Natl Acad Sci USA 2007 104 23 9667 9672 10.1073/pnas.0703820104 17535905 PMC1887587

[B9] Orom UA Nielsen FC Lund AH MicroRNA-10a binds the 5'UTR of ribosomal protein mRNAs and enhances their translation Mol Cell 2008 30 4 460 471 10.1016/j.molcel.2008.05.001 18498749

[B10] Forman JJ Legesse-Miller A Coller HA A search for conserved sequences in coding regions reveals that the let-7 microRNA targets Dicer within its coding sequence Proc Natl Acad Sci USA 2008 105 39 14879 14884 10.1073/pnas.0803230105 18812516 PMC2567461

[B11] Kloosterman WP Wienholds E Ketting RF Plasterk RH Substrate requirements for let-7 function in the developing zebrafish embryo Nucleic Acids Res 2004 32 21 6284 6291 10.1093/nar/gkh968 15585662 PMC535676

[B12] Lee I Ajay SS Yook JI Kim HS Hong SH Kim NH Dhanasekaran SM Chinnaiyan AM Athey BD New class of microRNA targets containing simultaneous 5'-UTR and 3'-UTR interaction sites Genome Res 2009 19 7 1175 1183 10.1101/gr.089367.108 19336450 PMC2704433

[B13] Kozak M Pushing the limits of the scanning mechanism for initiation of translation Gene 2002 299 1-2 1 34 10.1016/S0378-1119(02)01056-9 12459250 PMC7126118

[B14] Kozak M Structural features in eukaryotic mRNAs that modulate the initiation of translation J Biol Chem 1991 266 30 19867 19870 1939050

[B15] Avni D Biberman Y Meyuhas O The 5' terminal oligopyrimidine tract confers translational control on TOP mRNAs in a cell type- and sequence context-dependent manner Nucleic Acids Res 1997 25 5 995 1001 10.1093/nar/25.5.995 9023110 PMC146534

[B16] Iacono M Mignone F Pesole G uAUG and uORFs in human and rodent 5'untranslated mRNAs Gene 2005 349 97 105 10.1016/j.gene.2004.11.041 15777708

[B17] Churbanov A Rogozin IB Babenko VN Ali H Koonin EV Evolutionary conservation suggests a regulatory function of AUG triplets in 5'-UTRs of eukaryotic genes Nucleic Acids Res 2005 33 17 5512 5520 10.1093/nar/gki847 16186132 PMC1236974

[B18] Morris DR Geballe AP Upstream open reading frames as regulators of mRNA translation Mol Cell Biol 2000 20 23 8635 8642 10.1128/MCB.20.23.8635-8642.2000 11073965 PMC86464

[B19] Imataka H Nakayama K Yasumoto K Mizuno A Fujii-Kuriyama Y Hayami M Cell-specific translational control of transcription factor BTEB expression. The role of an upstream AUG in the 5'-untranslated region J Biol Chem 1994 269 32 20668 20673 8051167

[B20] Jin X Turcott E Englehardt S Mize GJ Morris DR The two upstream open reading frames of oncogene mdm2 have different translational regulatory properties J Biol Chem 2003 278 28 25716 25721 10.1074/jbc.M300316200 12730202

[B21] Kozak M An analysis of vertebrate mRNA sequences: intimations of translational control J Cell Biol 1991 115 4 887 903 10.1083/jcb.115.4.887 1955461 PMC2289952

[B22] Raney A Baron AC Mize GJ Law GL Morris DR In vitro translation of the upstream open reading frame in the mammalian mRNA encoding S-adenosylmethionine decarboxylase J Biol Chem 2000 275 32 24444 24450 10.1074/jbc.M003364200 10829027

[B23] Wang L Wessler SR Inefficient reinitiation is responsible for upstream open reading frame-mediated translational repression of the maize R gene Plant Cell 1998 10 10 1733 1746 10.1105/tpc.10.10.1733 9761799 PMC143946

[B24] Kwon HS Lee DK Lee JJ Edenberg HJ Ahn YH Hur MW Posttranscriptional regulation of human ADH5/FDH and Myf6 gene expression by upstream AUG codons Arch Biochem Biophys 2001 386 2 163 171 10.1006/abbi.2000.2205 11368338

[B25] Song KY Hwang CK Kim CS Choi HS Law PY Wei LN Loh HH Translational repression of mouse mu opioid receptor expression via leaky scanning Nucleic Acids Res 2007 35 5 1501 1513 10.1093/nar/gkm034 17284463 PMC1865057

[B26] Nikolcheva T Pyronnet S Chou SY Sonenberg N Song A Clayberger C Krensky AM A translational rheostat for RFLAT-1 regulates RANTES expression in T lymphocytes J Clin Invest 2002 110 1 119 126 12093895 10.1172/JCI15336 PMC151028

[B27] Griffiths-Jones S Saini HK van Dongen S Enright AJ miRBase: tools for microRNA genomics Nucleic Acids Res 2008 36 Database D154 158 17991681 10.1093/nar/gkm952 PMC2238936

[B28] Rehmsmeier M Steffen P Hochsmann M Giegerich R Fast and effective prediction of microRNA/target duplexes Rna 2004 10 10 1507 1517 10.1261/rna.5248604 15383676 PMC1370637

[B29] Xie X Lu J Kulbokas EJ Golub TR Mootha V Lindblad-Toh K Lander ES Kellis M Systematic discovery of regulatory motifs in human promoters and 3' UTRs by comparison of several mammals Nature 2005 434 7031 338 345 10.1038/nature03441 15735639 PMC2923337

[B30] Krek A Grun D Poy MN Wolf R Rosenberg L Epstein EJ MacMenamin P da Piedade I Gunsalus KC Stoffel M Combinatorial microRNA target predictions Nat Genet 2005 37 5 495 500 10.1038/ng1536 15806104

[B31] Bieker JJ Kruppel-like factors: three fingers in many pies J Biol Chem 2001 276 37 34355 34358 10.1074/jbc.R100043200 11443140

[B32] Black AR Black JD Azizkhan-Clifford J Sp1 and kruppel-like factor family of transcription factors in cell growth regulation and cancer J Cell Physiol 2001 188 2 143 160 10.1002/jcp.1111 11424081

[B33] Song A Nikolcheva T Krensky AM Transcriptional regulation of RANTES expression in T lymphocytes Immunol Rev 2000 177 236 245 10.1034/j.1600-065X.2000.17610.x 11138780

[B34] Kim J Inoue K Ishii J Vanti WB Voronov SV Murchison E Hannon G Abeliovich A A MicroRNA feedback circuit in midbrain dopamine neurons Science 2007 317 5842 1220 1224 10.1126/science.1140481 17761882 PMC2782470

[B35] Martinez NJ Ow MC Barrasa MI Hammell M Sequerra R Doucette-Stamm L Roth FP Ambros VR Walhout AJ A C. elegans genome-scale microRNA network contains composite feedback motifs with high flux capacity Genes Dev 2008 22 18 2535 2549 10.1101/gad.1678608 18794350 PMC2546694

[B36] Wang B Yanez A Novina CD MicroRNA-repressed mRNAs contain 40S but not 60S components Proc Natl Acad Sci USA 2008 105 14 5343 5348 10.1073/pnas.0801102105 18390669 PMC2291078

[B37] Gaba A Wang Z Krishnamoorthy T Hinnebusch AG Sachs MS Physical evidence for distinct mechanisms of translational control by upstream open reading frames Embo J 2001 20 22 6453 6463 10.1093/emboj/20.22.6453 11707416 PMC125715

[B38] Peters L Meister G Argonaute proteins: mediators of RNA silencing Mol Cell 2007 26 5 611 623 10.1016/j.molcel.2007.05.001 17560368

[B39] Tolia NH Joshua-Tor L Slicer and the argonautes Nat Chem Biol 2007 3 1 36 43 10.1038/nchembio848 17173028

[B40] Maere S Heymans K Kuiper M BiNGO: a Cytoscape plugin to assess overrepresentation of gene ontology categories in biological networks Bioinformatics 2005 21 16 3448 3449 10.1093/bioinformatics/bti551 15972284

[B41] Landgraf P Rusu M Sheridan R Sewer A Iovino N Aravin A Pfeffer S Rice A Kamphorst AO Landthaler M A mammalian microRNA expression atlas based on small RNA library sequencing Cell 2007 129 7 1401 1414 10.1016/j.cell.2007.04.040 17604727 PMC2681231

[B42] Chen J Lozach J Garcia EW Barnes B Luo S Mikoulitch I Zhou L Schroth G Fan JB Highly sensitive and specific microRNA expression profiling using BeadArray technology Nucleic Acids Res 2008 36 14 e87 10.1093/nar/gkn387 18579563 PMC2504321

[B43] Hohjoh H Fukushima T Marked change in microRNA expression during neuronal differentiation of human teratocarcinoma NTera2D1 and mouse embryonal carcinoma P19 cells Biochem Biophys Res Commun 2007 362 2 360 367 10.1016/j.bbrc.2007.07.189 17716626

[B44] Lawrie CH Saunders NJ Soneji S Palazzo S Dunlop HM Cooper CD Brown PJ Troussard X Mossafa H Enver T MicroRNA expression in lymphocyte development and malignancy Leukemia 2008 22 7 1440 1446 10.1038/sj.leu.2405083 18185523

[B45] Takada S Berezikov E Yamashita Y Lagos-Quintana M Kloosterman WP Enomoto M Hatanaka H Fujiwara S Watanabe H Soda M Mouse microRNA profiles determined with a new and sensitive cloning method Nucleic Acids Res 2006 34 17 e115 10.1093/nar/gkl653 16973894 PMC1635289

[B46] Jousse C Bruhat A Carraro V Urano F Ferrara M Ron D Fafournoux P Inhibition of CHOP translation by a peptide encoded by an open reading frame localized in the chop 5'UTR Nucleic Acids Res 2001 29 21 4341 4351 10.1093/nar/29.21.4341 11691921 PMC60176

