# Supplementary material for: Unified translation repression mechanism for microRNAs and upstream AUGs
Source: BMC Genomics. 2010 Mar 5;11:155. doi: 10.1186/1471-2164-11-155 (PMC2842251; doi:10.1186/1471-2164-11-155)
Supplement: Additional file 1 — Genes containing uAUGs that do/do not interact with 3'-ends of conserved miRNAs. GO-term analysis for two categories of genes that contain uAUGs. The first category consists of genes with uAUGs that are predicted to interact with 3'-ends of conserved miRNAs (likely targets). The second category of genes contains uAUGs but shows no such interactions. [file 1471-2164-11-155-S1.PDF]

GO-term analysis for genes that contain uAUGs listed in Table 1 using BiNGO (Maere *et al.*)

A1CF
ABCF2
ABCG1
ACCN1
ACTL6B
ACTR1A
ACVR2A
ADC
ADCK1
ADIPOR1
ADRA1B
ADRA2C
ADRM1
AFTPH
AGBL5
AHSA1
AJAP1
AKR1B10
ALDOA
ALX1
AMD1
AMFR
AMPH
ANKFY1
ANKH
ANKS1A
ANP32A
ANP32B
ANP32E
AP1G1
ARF1
ARF6
ARHGEF12
ARHGEF9
ARID4A
ARL15
ARL4C
ARL5A
ARL8A
ARNTL
ARPC4
ARPC5
ASB8
ASCC2
ASH1L
ASPH
ATAD2B
ATF4
ATF7
ATG5
ATG9A
ATOH8
ATP10A
ATP2A2
ATP2C1
ATP8A2
ATXN1
ATXN3
ATXN7
AZIN1
B3GALNT2
B3GALT2
B4GALT2
BACH2
BAI1
BAIAP2
BAIAP2L2
BANF1
BAP1
BARHL1
BARHL2
BAT1
BBC3
BBX
BCL11A
BCL11B
BCL3
BCL6
BCL7C
BCOR
BDNF
BDP1
BHLHB3
BHLHB5
BMP2
BMP2K
BMP6
BMPER
BRD2
BRD4
BTBD10
BTG1
C13orf7
C14orf100
C14orf147
C15orf41
C18orf1
C1orf119
C1orf164
C1orf25
C1orf27
C1orf76
C1QL2
C20orf24
C20orf67
C2orf25
C2orf33
C3orf10
C4orf18
C5orf41
C5orf5
CA10
CABIN1
CABYR
CACNA1E
CACNA1G
CACNA2D2
CACNG3
CADM1
CALB2
CALU
CAMK2B
CAMK2G
CAPRIN1
CASD1
CASK
CASP8AP2
CBLL1
CBX4
CBX6
CBX7
CBX8
CCDC109A
CCDC53
CCNB2
CCND1
CCNI
CCNJ
CD2AP
CD37
CDC2L1
CDH24
CDH8
CDK5RAP3
CDX1
CDYL2
CENTA2
CENTD1
CFL1
CHD2
CHD4
CHMP7
CHST11
CHSY1
CHUK
CITED2
CKAP5
CLCF1
CLCN3
CLCN5
CLDN23
CLDND1
CLIC1
CMTM4
CNIH2
CNKSR2
CNOT4
CNOT7
CNTN4
CNTN6
COIL
COL1A1
COL3A1
COL4A1
COLQ
COPS3
COPS4
CPEB3
CPSF3
CRABP1
CRBN
CREB1
CREB3L2
CREBL2
CRK
CRKRS
CRYAB
CRYGC
CSDE1
CSK
CSMD3
CSNK1D
CSNK2A1
CSNK2A2
CTDSP1
CTDSPL2
CTNNA2
CTNNBIP1
CTNND1
CUL5
CUX1
CXorf6
CYBA
DAB1
DCTN2
DDEF2
DDX25
DGCR2
DGCR8
DGKI
DGKZ
DHCR24
DICER1
DLX1
DLX3
DLX5
DNAJA2
DNAJB12
DNAJB5
DOC2A
DOC2B
DOK5
DOK6
DOK7
DOLPP1
DOT1L
DPP4
DPYSL2
DRP2
DSCAM
DSG1
DTNA
DTX3
DULLARD
DUSP15
DUSP16
DUSP6
DVL3
DYRK1A
DYRK1B
EBF1
EDA
EDC4
EEF1D
EEF1G
EFNA1
EFNA3
EFNA5
EFNB1
EFTUD2
EGLN2
EHBP1
EIF1
EIF1B
EIF2S2
EIF3B
EIF4A2
EIF4G2
EIF4G3
EIF4H
EIF5A
EIF5A2
ELAVL1
ELAVL2
ELK3
ELL2
ELOVL1
ELOVL6
EML5
EMX2
EN1
ENAH
ENTPD7

EPC1	*FOXA1*	*HAND1*	*HS6ST1*	*KCNN3*	*LEPROTL1*	*MED31*
EPHA3	*FOXG1*	*HCCA2*	*HS6ST3*	*KCNN4*	*LGI1*	*MED7*
EPHA4	*FOXJ1*	*HCN2*	*HSD11B2*	*KCNS2*	*LHX5*	*MEF2C*
EPHB2	*FOXJ3*	*HDAC2*	*HSDL1*	*KCNS3*	*LIN28*	*MEIS1*
ERF	*FOXN3*	*HDAC4*	*HSP90AB1*	*KCTD10*	*LMO1*	*MEMO1*
ERGIC3	*FRS2*	*HDAC5*	*ICK*	*KCTD15*	*LMO2*	*MEN1*
ERRFI1	*FRS3*	*HDGFRP3*	*ID2*	*KCTD17*	*LMO4*	*METTL3*
ETF1	*FURIN*	*HEATR3*	*ID3*	*KHDRBS3*	*LOXL1*	*MEX3C*
ETV1	*FYN*	*HELZ*	*IHPK1*	*KHK*	*LRFN2*	*MFSD2*
ETV2	*G3BP1*	*HERC4*	*IKZF1*	*KIAA0082*	*LRP2*	*MGAT2*
ETV5	*GABRB2*	*HERPUD2*	*IL1RAPL1*	*KIAA0427*	*LRRC4*	*MGAT3*
EXOC1	*GABRG2*	*HES1*	*IL7*	*KIAA0562*	*LRRC4C*	*MGAT4B*
EXOC5	*GADD45A*	*HEXIM1*	*ILK*	*KIAA1219*	*LRRTM3*	*MGC4172*
EXT1	*GALNT7*	*HGS*	*ING3*	*KIAA1715*	*LRRTM4*	*MIDN*
EYA1	*GATAD2B*	*HHIP*	*INHBA*	*KIF3C*	*LTBP1*	*MIER1*
FA2H	*GDF5*	*HIP1R*	*INHBB*	*KIF5B*	*LUC7L*	*MINK1*
FAF1	*GGNBP2*	*HIP2*	*INPP5A*	*KIT*	*LYAR*	*MKRN1*
FAM110B	*GJB1*	*HMBOX1*	*IPO13*	*KITLG*	*MAFB*	*MLF2*
FAM129A	*GLIS3*	*HMG2L1*	*IRF2*	*KLF12*	*MAGI3*	*MLL5*
FAM33A	*GNA11*	*HMGB1*	*IVNS1ABP*	*KLF13*	*Magmas*	*MLLT3*
FAM70A	*GNAI2*	*HMGB3*	*JAK1*	*KLF4*	*MAML3*	*MME*
FAM98A	*GNAI3*	*HNRNPC*	*JAKMIP2*	*KLF7*	*MAP2K2*	*MNT*
FBXL3	*GNAT1*	*HNRNPR*	*JARID2*	*KLF9*	*MAP3K11*	*MORF4L1*
FBXL4	*GNAZ*	*HNRPAB*	*JAZF1*	*KLHL10*	*MAP3K2*	*MOSPD1*
FBXO42	*GOLIM4*	*HOXA1*	*JDP2*	*KLHL18*	*MAP3K7IP2*	*MOSPD3*
FBXW11	*GPBP1*	*HOXA11*	*JMJD1A*	*KLHL20*	*MAPK1*	*MPP5*
FBXW2	*GPD1L*	*HOXA3*	*JMJD1C*	*KLHL24*	*MAPK10*	*MRVI1*
FEM1B	*GPHN*	*HOXA4*	*JPH1*	*KLHL28*	*MAPK8IP3*	*MSL3L1*
FEN1	*GPR26*	*HOXA9*	*JPH4*	*KPNA3*	*MAPKAP1*	*MSX1*
FEV	*GPR61*	*HOXB13*	*JUN*	*KPNA4*	*MARCKS*	*MTA1*
FEZF2	*GPR85*	*HOXB3*	*KATNB1*	*KRTAP4-5*	*MAST1*	*MTCP1*
FGD1	*GPSM2*	*HOXB6*	*KBTBD4*	*LAD1*	*MATR3*	*MTMR14*
FGD6	*GRIA2*	*HOXC13*	*KBTBD8*	*LASS6*	*MAX*	*MTPN*
FGF14	*GRIA3*	*HOXC6*	*KCNA4*	*LBX1*	*MBD2*	*MYBL2*
FGF8	*GRID1*	*HOXC8*	*KCNB1*	*LCE1B*	*MBNL1*	*MYBPC1*
FGFR1	*GRIN2A*	*HOXD4*	*KCND2*	*LCE1E*	*MBNL2*	*MYC*
FIS1	*GRIPAP1*	*HOXD9*	*KCNH2*	*LCE3D*	*MBOAT2*	*MYL3*
FLII	*GRK1*	*HPCA*	*KCNIP1*	*LCORL*	*MBTD1*	*MYST2*
FLRT3	*GSC*	*HR*	*KCNJ2*	*LDB1*	*MBTPS1*	*MYST3*
FMO1	*GTF3C2*	*HRB*	*KCNJ8*	*LDB2*	*MCF2*	*MYST4*
FMR1	*GTPBP1*	*HS2ST1*	*KCNK3*	*LDLRAP1*	*MCRS1*	*MYT1*

NAP1L1	*NUTF2*	*PGM5*	*PPAP2A*	*RAB13*	*RHOB*	*SENP2*
NAT12	*NXN*	*PGRMC1*	*PPAPDC3*	*RAB14*	*RHOBTB2*	*SERP1*
NBR1	*OAZ2*	*PH-4*	*PPARGC1A*	*RAB1A*	*RHOG*	*SERTAD2*
NCK2	*ODF2*	*PHACTR1*	*PPFIA2*	*RAB2A*	*RIC8B*	*SF3B14*
NCOA2	*OPA3*	*PHACTR3*	*PPM1A*	*RAB31*	*RICH2*	*SFN*
NDEL1	*ORMDL2*	*PHEX*	*PPM1B*	*RAB33A*	*RLBP1L1*	*SFRS15*
NDUFAB1	*OTUB1*	*PHF1*	*PPM1D*	*RAB35*	*RND3*	*SFRS16*
NEK11	*PACS1*	*PHF10*	*PPM1G*	*RAB39B*	*RNF10*	*SFRS9*
NEK6	*PAFAH1B1*	*PHF12*	*PPP1R10*	*RAB5A*	*RNF126*	*SH3GL1*
NELF	*PAK1*	*PHF2*	*PPP1R16A*	*RAB6A*	*RNF139*	*SH3GLB2*
NEUROD6	*PAK3*	*PHF21A*	*PPP1R7*	*RAC1*	*RNF144A*	*SH3RF1*
NF1	*PAPOLG*	*PHF21B*	*PPP2CA*	*RAC3*	*RNF41*	*SH3RF2*
NF2	*PARD6A*	*PHF23*	*PPP2CB*	*RAD23A*	*RNPEPL1*	*SIAH2*
NFAT5	*PARK2*	*PHF3*	*PPP2R2B*	*RAD50*	*ROD1*	*SIRPA*
NFATC3	*PARP6*	*PHOX2A*	*PPP2R5E*	*RAI1*	*RORC*	*SIX2*
NFATC4	*PARP8*	*PHTF1*	*PPP3CA*	*RALBP1*	*RPIA*	*SIX3*
NFE2L1	*PAX3*	*PI4K2A*	*PPP3CB*	*RALGPS2*	*RPL12*	*SKP1*
NFIA	*PAX6*	*PIAS1*	*PPP4C*	*RALYL*	*RPL13*	*SLC20A1*
NFIB	*PBRM1*	*PIAS3*	*PPP4R1L*	*RANBP9*	*RPL21*	*SLC25A36*
NFIX	*PBX1*	*PIAS4*	*PRKCA*	*RAP2A*	*RPL29*	*SLC25A5*
NKD1	*PBX3*	*PICALM*	*PRKCE*	*RARB*	*RPLP0*	*SLC26A9*
NKIRAS2	*PCBP2*	*PIK3CG*	*PRKD3*	*RASD1*	*RPP25*	*SLC35A4*
NKX2-8	*PCDH7*	*PIK3R3*	*PRKG1*	*RASGRP1*	*RPS2*	*SLC35E1*
NLGN3	*PCGF1*	*PITX1*	*PROX1*	*RAX*	*RPS6KA1*	*SLC39A13*
NLK	*PCGF2*	*PKIG*	*PRPH2*	*RBBP5*	*RTN2*	*SLC41A1*
NOC3L	*PCSK1N*	*PLAG1*	*PRRX1*	*RBM10*	*RUNX1T1*	*SLC43A2*
NR1D1	*PCTK1*	*PLCB1*	*PSCD2*	*RBM12*	*RUSC1*	*SLC4A10*
NR2C2	*PCYT1B*	*PLCH1*	*PSKH1*	*RBM12B*	*RXRA*	*SLC4A3*
NR4A2	*PDAP1*	*PLD5*	*PSMB3*	*RBM18*	*RXRG*	*SLC6A8*
NRBP1	*PDCD10*	*PLEKHA2*	*PSMC4*	*RBM39*	*RYBP*	*SLCO3A1*
NRIP1	*PDE1B*	*PLK3*	*PSME3*	*RCAN2*	*SATB2*	*SMAD5*
NRN1	*PDE7B*	*PLP1*	*PTEN*	*REEP1*	*SBDS*	*SMAD6*
NRSN1	*PDGFA*	*PLSCR3*	*PTGES3*	*REEP5*	*SBF1*	*SMAD7*
NRXN2	*PDGFC*	*PNKD*	*PTP4A1*	*RELA*	*SCAMP1*	*SMCR7L*
NRXN3	*PDPK1*	*PNRC2*	*PTPLAD1*	*RELL2*	*SCARF2*	*SMURF1*
NTF5	*PDXDC1*	*POLR3F*	*PTPN23*	*REPS2*	*SCN3A*	*SMURF2*
NTNG1	*PDZD2*	*PORCN*	*PTPRK*	*RER1*	*SCN5A*	*SNCA*
NTRK3	*PELI2*	*POU2F1*	*PUM2*	*REV1*	*SCYL1*	*SNCAIP*
NUDT3	*PEX11B*	*POU2F2*	*PVRL4*	*RFTN2*	*SELI*	*SNCB*
NUMBL	*PEX5*	*POU4F2*	*PYGO2*	*RGL2*	*SEMA4G*	*SND1*
NUS1	*PFN1*	*POU6F2*	*RAB10*	*RHOA*	*SEMA7A*	*SNF1LK2*

SNIP	*STX1B*	*TLL1*	*TRIM62*	*UBE2Q1*	*VWC2*	*ZDHHC5*
SNN	*SUMO2*	*TLOC1*	*TRIM66*	*UBE2R2*	*WAC*	*ZFAND3*
SNRPB	*SURF4*	*TMCO6*	*TRIM8*	*UBE4A*	*WAPAL*	*ZFAND5*
SNRPD1	*SUSD4*	*TMEFF2*	*TRIOBP*	*UBE4B*	*WASF2*	*ZFAND6*
SNRPD2	*SUV420H1*	*TMEM1*	*TRPM3*	*UBL7*	*WDR44*	*ZFHX3*
SNRPD3	*SV2A*	*TMEM121*	*TRPS1*	*UBOX5*	*WNT10A*	*ZFHX4*
SNUPN	*SYNCRIP*	*TMEM161B*	*TSC1*	*UBP1*	*WNT11*	*ZFP161*
SNX24	*SYT13*	*TMEM49*	*TSC22D1*	*UBQLN2*	*WNT2*	*ZFR*
SNX5	*TAF10*	*TMOD4*	*TSC22D3*	*UBTD1*	*WNT9A*	*ZFYVE27*
SOCS3	*TAF5L*	*TMSB10*	*TSC22D4*	*UCHL3*	*WRNIP1*	*ZIC1*
SORCS3	*TAF9*	*TMTC2*	*TSHZ2*	*UCK2*	*WSB1*	*ZIC3*
SOX21	*TAGLN3*	*TNFAIP1*	*TSPAN12*	*UGCG*	*WSCD1*	*ZIC5*
SOX4	*TAOK2*	*TNFRSF11A*	*TSPAN17*	*UPF2*	*WWP1*	*ZMIZ1*
SP1	*TBC1D15*	*TNFSF8*	*TSPAN18*	*USF1*	*XPO1*	*ZMYND11*
SP4	*TBL1XR1*	*TNMD*	*TSPAN5*	*USF2*	*XPR1*	*ZNF148*
SPA17	*TBPL1*	*TNPO1*	*TSPAN9*	*USP12*	*YES1*	*ZNF219*
SPAG7	*TBR1*	*TNPO2*	*TSSK6*	*USP19*	*YIPF4*	*ZNF238*
SPI1	*TBX2*	*TNRC4*	*TTC9B*	*USP2*	*YWHAE*	*ZNF282*
SRPK1	*TBX3*	*TOLLIP*	*TTLL11*	*USP42*	*YWHAQ*	*ZNF318*
SRRM1	*TCEB2*	*TOX4*	*TTLL5*	*USP46*	*YY1*	*ZNF32*
SSBP2	*TCF7L2*	*TPD52L2*	*TULP4*	*USP48*	*ZBTB2*	*ZNF410*
SSNA1	*TEAD1*	*TPM1*	*TWIST1*	*USP49*	*ZBTB20*	*ZNF491*
ST3GAL3	*TEAD2*	*TPM2*	*TWIST2*	*USP52*	*ZBTB33*	*ZNF503*
ST5	*TEK*	*TRA2A*	*TXNIP*	*VANGL2*	*ZBTB7B*	*ZNF521*
ST6GALNAC5	*TESK1*	*TRAF4*	*TYRO3*	*VAPA*	*ZC3H10*	*ZNF532*
ST6GALNAC6	*TFAP2E*	*TRAM1*	*UBA1*	*VEGFA*	*ZC3H15*	*ZNF592*
ST8SIA2	*TFE3*	*TRIM11*	*UBAC1*	*VEZF1*	*ZC3H7B*	*ZNF627*
ST8SIA3	*THAP1*	*TRIM2*	*UBAP1*	*VPS26B*	*ZCCHC14*	*ZNF638*
ST8SIA4	*THOC4*	*TRIM3*	*UBAP2L*	*VPS33B*	*ZCCHC17*	*ZNF710*
STAG1	*THRA*	*TRIM33*	*UBE2E1*	*VPS36*	*ZCCHC6*	*ZNF804A*
STK36	*TIA1*	*TRIM37*	*UBE2E2*	*VPS4A*	*ZDHHC14*	*ZNRF1*
STRN3	*TLE4*	*TRIM46*	*UBE2M*	*VSX2*	*ZDHHC3*	*ZZZ3*

Number of genes among 1071 (above) with available annotations = Number of genes in test set (X) = 678
Number of genes in reference set (N) = 8649
x indicates the number of genes in test set in the functional category listed
n indicates the number of genes in reference set in the functional category listed
p-values calculated using hypergeometric test.

GO-ID	p-value	corr p-value	x	n	X	N	Description
30528	1.1933E-17	7.6612E-15	145	923	678	8649	transcription regulator activity
3700	5.5821E-11	1.7919E-8	84	520	678	8649	transcription factor activity
16564	4.0031E-10	8.5667E-8	39	168	678	8649	transcription repressor activity
5488	3.7291E-9	5.9853E-7	527	5881	678	8649	binding
8134	1.9686E-8	2.5155E-6	59	356	678	8649	transcription factor binding
3676	2.3510E-8	2.5155E-6	138	1132	678	8649	nucleic acid binding
3702	4.2668E-7	3.9133E-5	36	189	678	8649	RNA polymerase II transcription factor activity
3677	6.9153E-7	5.5495E-5	104	836	678	8649	DNA binding
3924	1.5696E-6	1.1197E-4	27	128	678	8649	GTPase activity
5515	2.0281E-6	1.3020E-4	430	4753	678	8649	protein binding
4722	1.2510E-5	7.3011E-4	11	31	678	8649	protein serine/threonine phosphatase activity
16462	1.7399E-5	9.3085E-4	42	274	678	8649	pyrophosphatase activity
16817	2.0819E-5	9.5472E-4	42	276	678	8649	hydrolase activity, acting on acid anhydrides
16818	2.0819E-5	9.5472E-4	42	276	678	8649	hydrolase activity, acting on acid anhydrides, in phosphorus-containing anhydrides
17111	2.3087E-5	9.8813E-4	40	259	678	8649	nucleoside-triphosphatase activity
3712	3.3140E-5	1.3298E-3	40	263	678	8649	transcription cofactor activity
3714	4.9684E-5	1.8763E-3	20	97	678	8649	transcription corepressor activity
4674	1.3454E-3	4.7985E-2	30	215	678	8649	protein serine/threonine kinase activity

GO-term analysis for genes containing uAUG sequences that do not exhibit Watson-Crick interactions with 3′-ends of conserved miRNAs (Total 716)

ABI2	*ARPC2*	*C1orf77*	*CHD6*	*DDX6*	*ERH*	*GABRB1*
ABLIM1	*ASF1A*	*C20orf52*	*CHMP4B*	*DENND4A*	*ETS1*	*GALNT1*
ACSL4	*ASTN1*	*C5orf13*	*CITED1*	*DES*	*ETV3*	*GALNT5*
ACTR2	*ATAD1*	*C6orf62*	*CKS1B*	*DHX30*	*ETV6*	*GALNTL4*
ACTR3	*ATN1*	*C7orf23*	*CLPTM1*	*DHX40*	*EWSR1*	*GAP43*
ACTR6	*ATP11B*	*C9orf126*	*CNBP*	*DHX9*	*EXOC4*	*GARNL4*
ACVR1B	*ATP1B2*	*CABP7*	*CNN2*	*DIS3L*	*EXOSC1*	*GBF1*
ADAMTS10	*ATP2A1*	*CACNA2D1*	*CNOT10*	*DLG1*	*EZH2*	*GBX2*
AFF4	*ATP6V1C1*	*CACNB3*	*CNOT2*	*DLG3*	*FAM108B1*	*GDI2*
AHCYL1	*B3GAT1*	*CACNG2*	*CNOT3*	*DMRT3*	*FAM126B*	*GGA1*
AIFM1	*B3GAT3*	*CAMK1*	*CNOT6*	*DMTF1*	*FAM134A*	*GJC2*
AIP	*B4GALT5*	*CAMK4*	*CNOT6L*	*DNAJA4*	*FBLN5*	*GLCE*
AK3L1	*B4GALT7*	*CAMKK2*	*COL1A2*	*DNAJB4*	*FBXL2*	*GLRA2*
AKAP10	*BAI2*	*CAMTA2*	*COL4A3BP*	*DNAJC11*	*FBXL5*	*GLTP*
AKT3	*BCL2L1*	*CAPN6*	*COPG2*	*DNAJC6*	*FBXO36*	*GNA13*
ALDH9A1	*BCL2L11*	*CAPZA2*	*COPZ2*	*DOCK3*	*FBXO46*	*GNAQ*
ALX4	*BCL7A*	*CASC3*	*CPEB4*	*DPF2*	*FBXW7*	*GNB2*
AMBN	*BEX1*	*CCBL2*	*CPS1*	*DPH5*	*FGF12*	*GNB5*
AMELX	*BLCAP*	*CCDC130*	*CREBBP*	*DSCR3*	*FGF17*	*GNPDA2*
AMMECR1	*BMI1*	*CCDC55*	*CRISPLD1*	*DTD1*	*FGF9*	*GOLGA1*
ANKMY2	*BMP7*	*CCM2*	*CRY1*	*DYNC1LI1*	*FGFR1OP*	*GPC3*
ANKRD11	*BNC2*	*CCNG1*	*CSNK1G2*	*DYNC1LI2*	*FHIT*	*GRASP*
ANXA1	*BNIP2*	*CDC2L6*	*CSNK1G3*	*DYNLL2*	*FLI1*	*GRB2*
AP1S2	*BRD7*	*CDC42BPB*	*CTCF*	*E2F4*	*FLOT1*	*GRHL2*
AP2M1	*BRDT*	*CDC73*	*CTPS*	*EED*	*FLOT2*	*GRIK5*
AP3M1	*BTBD14A*	*CDGAP*	*CTR9*	*EFCBP2*	*FOSB*	*GRIN3A*
AP3S1	*BTF3*	*CDH11*	*CTTNBP2*	*EHD1*	*FOSL2*	*GRIP1*
APBB2	*BTG2*	*CDH13*	*CTTNBP2NL*	*EIF2C3*	*FOXB1*	*GRK5*
APBB3	*BUB3*	*CDH2*	*CUGBP1*	*EIF2S1*	*FOXD2*	*GSK3B*
APPL1	*BZW1*	*CDH7*	*CUL1*	*ELF1*	*FOXM1*	*GTPBP2*
ARHGAP30	*C11orf73*	*CDK5*	*CUL3*	*ENC1*	*FOXN2*	*H1F0*
ARHGEF7	*C12orf30*	*CDK8*	*CXCL14*	*ENOX2*	*FOXP2*	*H1FX*
ARL2	*C14orf4*	*CDKN2C*	*CXXC5*	*ENSA*	*FRAP1*	*H2AFZ*
ARL8B	*C14orf43*	*CEBPE*	*DAZAP2*	*EPB41L4A*	*FRMD5*	*HAS2*
ARMC8	*C17orf28*	*CENTG2*	*DCUN1D1*	*EPB41L4B*	*G3BP2*	*HBP1*
ARMCX3	*C1GALT1C1*	*CFL2*	*DDEF1*	*EPC2*	*GABRA1*	*HDGF*
ARPC1B	*C1orf58*	*CHCHD3*	*DDX3X*	*EPHA7*	*GABRA4*	*HDLBP*

HESX1
HGD
HIPK1
HIPK3
HIST2H2AA3
HMGB2
HNRNPA2B1
HNRPF
HNRPH2
HNRPM
HNT
HOMER1
HOMER2
HOXA10
HOXA2
HOXA5
HOXA7
HOXB4
HOXB5
HOXB7
HOXB8
HOXB9
HOXC11
HOXC5
HOXC9
HOXD10
HOXD3
HSF2
HSP90AA1
HSPA4
IBSP
IBTK
ID4
IFRD1
IGF2BP1
IGF2BP2
IGSF9
ILF2
IMMP2L
IMP3
ING4
INSM1
INTS12
IRX2
IRX4
ISL1
ISL2
ITGB8
ITPR1
KBTBD7
KCNA6
KCNC1
KCNC4
KCNG3
KCNH5
KCNJ3
KCNK1
KCNMB2
KCTD4
KDELR1
KDELR2
KIAA0494
KIAA1267
KIF13A
KIF2A
KIFAP3
KLHDC3
KPNA1
KRT16
L3MBTL3
LARP5
LATS2
LCOR
LDLRAD3
LENG1
LHFPL2
LHX1
LHX2
LIM2
LINGO2
LLGL1
LMBR1L
LMBRD2
LRFN5
LRP1
LRP1B
LRRC42
LRRC59
LRRC8D
LRRTM1
LZIC
MAB21L1
MAB21L2
MACROD1
MAF1
MAFG
MAML2
MAN1A1
MAP2K4
MAP2K6
MAP4K3
MAPK14
MAPK6
MAPK8
MAPK8IP2
MAPRE3
MARCH5
MARCKSL1
MARK3
MBD3
MCTS1
MDH1
MDM1
MECP2
MEF2D
METT10D
MFSD5
MGAT5
MGAT5B
MIER3
MIR16
MLLT10
MOBKL1A
MOBKL3
MORF4L2
MOV10
MRLC2
MSI1
MTCH2
MTDH
MYCBP
MYF6
MYLIP
MYNN
MYOT
NACA2
NALCN
NARG1
NAV1
NCAM1
NCAM2
NCOA5
NDP
NEDD8
NEK9
NEUROD2
NFATC1
NIT1
NKX2-1
NKX2-2
NMNAT2
NMT2
N-PAC
NPTXR
NR2E1
NR2F1
NR2F2
NRAS
NUP93
OAZ1
OAZ3
ODF1
OGDH
OLIG3
ONECUT2
OPHN1
OTP
OTUB2
OTX1
OTX2
OXSR1
PAIP2
PALMD
PAX9
PCDHB8
PCDHGC3
PCGF3
PCMT1
PCMTD1
PDE6D
PDIK1L
PDP2
PFN2
PHC3
PHF14
PHF17
PHF20
PHIP
PHOX2B
PITPNA
PJA2
POLG
POLR2J2
POLS
POU2AF1
PPARG
PPARGC1B
PPL
PPM1E
PPM1H
PPP1CA
PPP1CB
PPP2R2C
PPP2R4
PPP5C
PPRC1
PPTC7
PRKAA2
PRKACA
PRKCB1
PRKCI
PRPSAP2
PSIP1
PSMA1
PSMA7
PSMC6
PSMD11
PSMD14
PSMD3
PTBP2
PTP4A2
PTPN11
PTPN12
PTPN4
PTPRG
PUM1
PURA
PURB
PVRL1
QKI
RAB11A
RAB1B
RAB2B
RAB30
RAB6B
RAB8A
RABL3
RAF1
RANBP2
RAP2B
RAP2C
RASIP1
RBBP6
RBBP7
RBM35B
RBM4B
RBM5
RBPMS
RDH10
REEP2
RFC1
RGS3
RLF
RNF122
RNF20
RNPS1
RPL10A
RPL15
RPL27A
RPL30
RPL31
RPN2
RPS8
RRAGC
SAP18
SAPS3
SAR1A
SARS
SAT1
SATB1
SEMA6A
SEPT5
SERBP1
SFRS10
SFRS12
SFRS6
SGIP1
SGMS2
SH3BGRL
SH3BGRL3
SHC1
SHOC2
SIRT1
SIX1
SLC10A7
SLC12A5
SLC15A2

SLC22A4
SLC22A5
SLC25A13
SLC25A14
SLC35B4
SLC39A10
SLIT3
SLITRK2
SLITRK5
SLK
SMAD9
SMAP1
SMAP1L
SMARCC2
SMARCE1
SMYD5
SNRPA1
SNRPN
SNX8
SORBS2
SOX17
SOX18
SOX2
SOX6
SOX9
SPARC
SPATA6
SPOP
SPRY2
SPTLC2
SRF
SRGAP1
SRP54
SRPK2
SRR
SSBP3
STAG2
STC1
STK32B
STK39
STK40
STMN2
STOML2
STRBP
STT3A
STUB1
STX5
STX8
STXBP6
SUMO1
SUMO3
SUZ12
SYN2
SYT14
SYT9
TACC2
TAF12
TAF5
TAOK1
TBC1D19
TBC1D22B
TCEB3
TCF20
TCF4
TFAP2B
TFAP2C
TFAP2D
TFDP2
TGFB3
THAP7
TIAL1
TLK1
TMED1
TMEM108
TMEM135
TMEM164
TMEM185A
TMEM39B
TMEM59
TMEM60
TMEM93
TMSB4X
TNFAIP3
TOP1
TOX
TPH2
TPM3
TRAIP
TRAPPC3
TRAPPC6B
TRDN
TRIB2
TRIP4
TRPM7
TSN
TSPAN15
TUB
TUBB6
TUBG2
TUSC2
TXNDC11
U2AF1L4
UBE2D3
UBE2L3
UBE2N
UBE2S
UBE2V2
UBE3A
UBN1
UBXD8
UGP2
USP25
USP28
USP5
USP8
UTX
VCPIP1
VIM
VKORC1L1
VPRBP
VPS25
VPS26A
VPS54
VPS72
WBP2
WDFY3
WDR1
WDR48
WDR67
WDR68
WNT10B
WNT3
WNT7A
WNT7B
WT1
WWP2
XKR6
XPNPEP1
XPO7
XYLT1
YBX1
YTHDF2
YTHDF3
YWHAG
ZBTB1
ZBTB10
ZBTB12
ZBTB16
ZBTB26
ZBTB37
ZBTB39
ZBTB7A
ZCCHC5
ZDHHC23
ZEB1
ZEB2
ZHX1
ZMAT2
ZMYM3
ZMYND8
ZNF24
ZNF275
ZNF281
ZNF384
ZNF428
ZNF496
ZNF512
ZNF513
ZNF706
ZNF76

Number of genes among 716 (above) with available annotations = Number of genes in test set (X) = 448
Number of genes in reference set (N) = 8648
x indicates the number of genes in test set in the functional category listed
n indicates the number of genes in reference set in the functional category listed
p-values calculated using hypergeometric test.

GO-ID	p-value	corr p-value	x	n	X	N	Description	Genes in test set
30528	9.1528E-8	4.9059E-5	84	919	448	8648	transcription regulator activity	
5488	2.2855E-7	6.1252E-5	352	5879	448	8648	binding	
5515	3.5780E-7	6.3927E-5	297	4753	448	8648	protein binding	
3676	2.2083E-6	2.9592E-4	93	1131	448	8648	nucleic acid binding	
3700	1.2579E-5	1.3485E-3	50	519	448	8648	transcription factor activity	
3677	5.0034E-5	4.4697E-3	69	835	448	8648	DNA binding	
48027	1.3814E-4	1.0578E-2	3	3	448	8648	mRNA 5'-UTR binding	
3723	4.3415E-4	2.9088E-2	29	288	448	8648	RNA binding	
8134	7.5201E-4	4.0469E-2	33	355	448	8648	transcription factor binding	
3702	7.5501E-4	4.0469E-2	21	189	448	8648	RNA polymerase II transcription factor activity	
