# Supplementary material for: Unified translation repression mechanism for microRNAs and upstream AUGs
Source: BMC Genomics. 2010 Mar 5;11:155. doi: 10.1186/1471-2164-11-155 (PMC2842251; doi:10.1186/1471-2164-11-155)
Supplement: Additional file 2 — Predicted interactions between uAUG 6 and 7 (Table 5) of KLF9 and conserved miRNAs. uAUG6 and uAUG7 are thought to be responsible for limiting translation of KLF9 in HeLa cells but not in N2A. Predicted binding between both ends of conserved miRNAs in Table 5 and the two uAUGs are shown. [file 1471-2164-11-155-S2.PDF]

Predicted interactions between uAUG 6 and 7 (Table 5) of *KLF9* and conserved miRNAs

Shown below is a portion of the 5′-UTR of *KLF9* that contain uAUGs 6 and 7. The interactions and the associated free energy of binding were predicted using 11-mers and miRNA 5′- or 3′-ends. Full miRNAs and sequences surrounding the uAUGs are shown for clarity.

```
                          uAUG6                                     uAUG7
641 5′-UUUGGUUUGUGACGUGAUGGGAUUCUGCGAAAUUGUUACUGAGCAAGAGAAUGCCGGAACGUGCGGAC-3′ 708
```

```
target 5' G       UCUGCGA 3'       (uAUG6)
           AUGGGAU
           ||||||:            ΔG: -14.2 kcal/mol
           UACCCUG
miRNA  3'         UAGGAUGUAUACGUU 5' hsa-miR-448
```

```
target 5' C U       UUCUGCGA 3'   (uAUG6)
           G GAUGGGA
           | ||||:||       ΔG: -14.4 kcal/mol
           C CUACUCU
miRNA  3' U U       CUUUGUGGGA 5' hsa-miR-609
```

```
target 5' C          UCUG 3'       (uAUG6)
           GUGAUGGGAU
           ||:||||||:         ΔG: -16.5 kcal/mol
           CAUUACCCUG
miRNA  3'            GAAGGAGAAAC 5' hsa-miR-583
```

```
target 5'   C       GAUUCUGCGA 3'  (uAUG6)
             GUGAUGG
             |||||||       ΔG: -16.1 kcal/mol
             CACUACC
miRNA  3' UUC       AGUCGUCUGUAU 5'

                              hsa-miR-654-3p
```

```
target 5' UUGUGACGUG        C 3'   (uAUG6)
                    AUGGGAUU
                    ||||||||  ΔG: -14.7 kcal/mol
                    UACCCUAA
miRNA  3' UCUUCCGUGG        AU 5' hsa-miR-605
```

```
target 5' GAGA       AACGUGCG  3'  (uAUG7)
              AUGCCGG
              |||||:|      ΔG: -14.4 kcal/mol
              UACGGUC
miRNA  3' UCGA       GUAGAACGGA 5' hsa-miR-31
```
